# HMGB1: new biomarker and therapeutic target of autoimmune and autoinflammatory skin diseases

**DOI:** 10.3389/fimmu.2025.1569632

**Published:** 2025-04-16

**Authors:** Jinrong Fan, Kaiqiao He, Yonghui Zhang, Ruijing Li, Xiuli Yi, Shuli Li

**Affiliations:** ^1^ The College of Life Sciences, Northwest University, Xi’an, Shaanxi, China; ^2^ Department of Dermatology, Xijing Hospital, Fourth Military Medical University, Xi’an, Shaanxi, China

**Keywords:** HMGB1, autoimmune skin diseases, vitiligo, psoriasis, atopic dermatitis, alopecia areata

## Abstract

High-mobility group box 1 (HMGB1) is expressed in almost all human cells. During cell activation and cell death, the nucleoprotein HMGB1 can translocate to the extracellular space, thus mediating the early inflammatory response as an alarmin or damage-associated molecular pattern (DAMP). Extracellular HMGB1 interacts with immune cells by binding to pattern recognition Toll-like receptors (TLRs), including TLR2 and TLR4, and the receptor for advanced glycation end products (RAGE), thus mediating the immune response to protect the host against pathogens and maintain immune balance. HMGB1 is reportedly upregulated and is a critical biomarker for monitoring disease activity in several chronic inflammatory or autoimmune disorders, including multiple sclerosis, rheumatoid arthritis, inflammatory bowel disease, systemic lupus erythematosus and vitiligo. Additionally, the inhibition of HMGB1 expression or its activity has beneficial effects on disease activity in animal models of autoimmune diseases. Thus, HMGB1 is an indispensable biomarker and an important therapeutic target for autoimmune diseases. This review provides a detailed summary of the biological function of HMGB1 and provides a comprehensive outlook in terms of HMGB-focused diagnostic and therapeutic applications in autoimmune skin diseases.

## Introduction

High mobility group box 1 (HMGB1) is the most abundant member of the high mobility group (HMG) family of proteins and is expressed in almost all human cells (1). HMGB1 is located predominantly in the nucleus and can also actively and passively shuttle between the nucleus and the cytoplasm (2). During cell apoptosis and necrosis, HMGB1 is released from dying cells into the extracellular space (3). Extracellular HMGB1 functions as an alarmin or damage-associated molecular pattern (DAMP) and mediates immune cell migration to the site of tissue damage to protect against possible infection (1, 4). However, extracellular HMGB1 also functions as a proinflammatory cytokine that initiates an undesired immune response in a chronic inflammatory environment (5). Increasing numbers of studies have identified HMGB1 as an activity biomarker in various autoimmune diseases, including multiple sclerosis, rheumatoid arthritis, inflammatory bowel disease, systemic lupus erythematosus and vitiligo (6, 7). Research has shown that released HMGB1 binds to Toll-like receptors (TLRs) and receptor for advanced glycation products (RAGE), which are expressed on various cells, such as monocytes, macrophages, dendritic cells, T cells and B cells, and then activates the innate and/or adaptive immune response (8, 9). Recently, numerous studies have revealed that HMGB1 is a potential therapeutic target in several autoimmune diseases (10).

In this review, we summarize the biological functions and signalling mechanisms of HMGB1 in healthy individuals and patients with autoimmune skin diseases. Importantly, recent advances regarding the clinical relevance of HMGB1 and strategies to modify its release and biological activities for skin disease treatment are also discussed.

## The biological function of HMGB1

**Figure 1:**
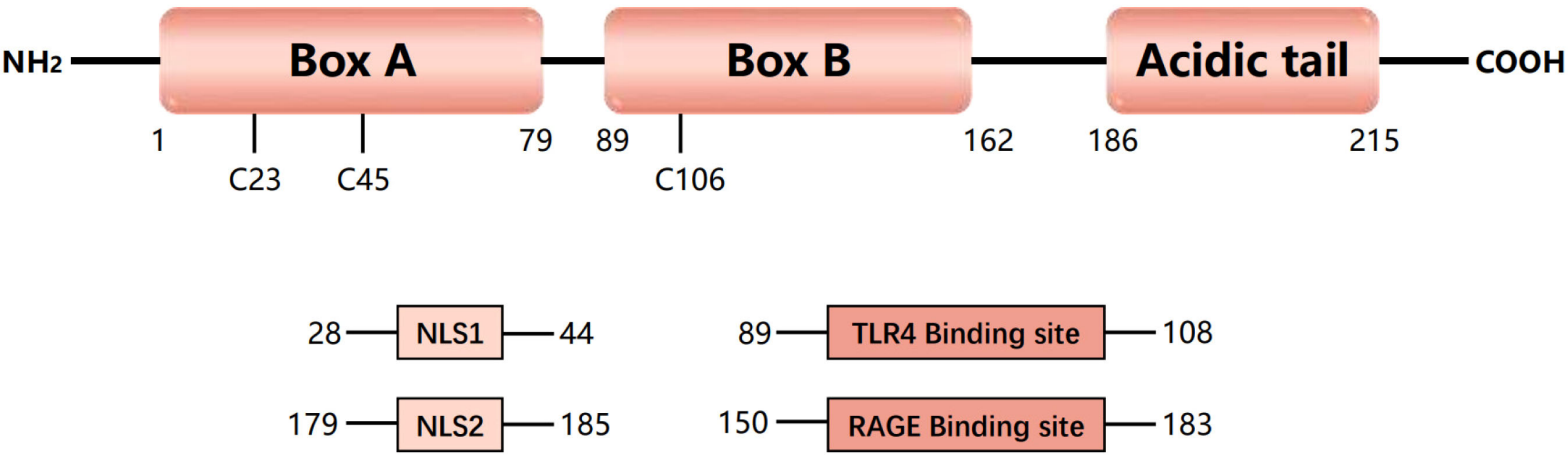
Structure and function of HMGB1. Human high-mobility group protein 1 (HMGB1) is an approximately 30 kDa protein composed of 215 amino acids. HMGB1 consists of three domains, including two DNA-binding domains (A-box and B-box) and a negatively charged C-terminal (acidic tail). HMGB1 contains three redox-sensitive cysteine residues (C23, C45, and C106). HMGB1 has two nuclear localization signals (NLS1 and NLS2), which are present mainly in the nucleus under normal physiological conditions. Nuclear HMGB1 is a DNA chaperone that maintains chromosome structure and function. Under oxidative stress, HMGB1 can act as a danger signal and can bind to its primary receptors, including Toll-like receptor 4 (TLR4) and receptor for advanced glycation end products (RAGE), to mediate an immune response.

HMGB1 was discovered nearly fifty decades ago (11). HMGB1 is a highly conserved nonhistone nucleoprotein 30 kDa in size with 99% homology in mammals (12). HMGB1 comprises 215 amino acid residues and contains two DNA-binding domains (A box domain and B box domain) and a C-terminal acidic tail (13) (**Figure 1**). HMGB1 plays an important role in development and reproduction, as embryonic global deletion of HMGB1 or conditional deletion of uterine HMGB1 leads to the death of mice shortly after birth (14, 15). The location of HMGB1 in the cell largely determines its function.

In most cells, HMGB1 is located in the nucleus and is highly expressed (16). As a DNA chaperone, nuclear HMGB1 recognizes specific DNA structures rather than specific DNA sequences (17). By binding DNA and histones, HMGB1 helps maintain the structure of nucleosomes, thereby preventing DNA damage (18). HMGB1 can also regulate the strength of histone and DNA interactions, thereby affecting the packaging of DNA into chromatin (19). By binding to small grooves of linear DNA and bending into helical structures, HMGB1 affects the accessibility of DNA to cellular factors involved in DNA replication, transcription, DNA repair, chromatin remodelling, and V(D)J recombination in T and B cells (20, 21). In mouse cells or tissues, the conditional depletion of HMGB1 leads to genomic instability, telomere shortening and nucleosome release, which results in inflammation and activates innate immunity (22). In an extremely rare genetic disease called congenital brachyphalangy and also in polydactyly and tibial aplasia/hypoplasia syndrome (BPTA syndrome), a frameshift variant in the acidic tail of HMGB1 alters HMGB1 phase separation and enhances its partitioning into the nucleolus, which results in nucleolar dysfunction and eventually, body development disorders (23). These findings provide direct evidence that nuclear HMGB1 maintains nuclear homeostasis and human development.

In addition to its nuclear location, some cells express HMGB1 in the cytoplasm or on the plasma membrane. In mouse fibroblasts and human cancer cells, cytosolic HMGB1 promotes autophagy by interacting with the autophagy core driver beclin 1 in response to various environmental stresses, such as starvation and oxidative damage (24). In lipopolysaccharide-treated brain microglia, HMGB1 competitively interacts with nucleotide binding oligomerization domain containing 2 (NOD2), thus inducing the formation of autophagosomes (25). Membrane-associated HMGB1, also referred to as amphoterin (26), is involved in the mediation of neurite outgrowth (27), smooth muscle cell chemotaxis (28) and tumour cell metastasis (29).

The post-translational modifications (PTMs) of HMGB1 are important for determining its function and release mechanisms (30). In addition, the proinflammatory cytokine activity of HMGB1 is closely related to the redox status of its three cysteine residues, which are mainly divided into reduced, disulfide, and oxidized forms. Reduced HMGB1 contains three cysteine residues (C23, C45, and C106), all of which are in the reduced state (-SH). Reduced HMGB1 primarily has chemotactic activity to attract immune cells to sites of injury or infection (31). In contrast, in disulfide-containing HMGB1, C23 and C45 form intramolecular disulfide bonds (C23-S-S-C45), whereas C106 remains in the reduced state. By binding to the TLR4 receptor, HMGB1 disulfide activates NF-κB signalling pathway, induces the production of cytokines and chemokines, and participates in the inflammatory response and the immune response (32, 33). In contrast, fully oxidized HMGB1 does not exert a proinflammatory effect.

## HMGB1 in autoimmune and autoinflammatory skin diseases

The role of HMGB1 in skin diseases has been extensively studied over the past 20 years (34). Increased levels of HMGB1 and increased numbers of HMGB1-secreting cells have been identified in skin lesions, including in vitiligo, psoriasis, atopic dermatitis, and pemphigus. Here, we discuss the role and new insights of HMGB1 in these skin diseases.

## HMGB1 in vitiligo

Vitiligo is a cutaneous depigmentation disorder characterized by the selective loss of melanocytes. Genetic predisposition, oxidative stress, and the autoimmune response have been implicated in the loss of functional melanocytes in vitiligo. Recent data have shown that HMGB1 is overexpressed in both blood and lesional samples from vitiligo patients (7, 35, 36).

Due to the genetic background of vitiligo patients, melanocytes are very sensitive to oxidative stress and ultraviolet radiation and thus they are prone to damage and release HMGB1 (35). In ultraviolet radiation B (UVB)-exposed melanocytes, nuclear factor-κB (NF-κB) and Janus Kinase (JAK) signalling have been implicated in HMGB1 translocation and release; by binding to RAGE in melanocytes, extracellular HMGB1 functions in the activation of downstream JAK1, JAK2, signal transducer and activator of transcription 1 (STAT1) and phosphorylated-extracellular signal-regulated kinase 1/2 (p-ERK1/2) signalling and in the mediation of the UVB-induced senescence-associated secretory phenotype as well as resistance to melanocyte cell death (37). In addition to these signalling pathways, autophagy can regulate the trafficking, secretion and degradation of HMGB1 in several cell types (1, 38). Previous studies have revealed that melanocytes in vitiligo exhibit dysregulated autophagy and hypersensitivity to oxidative injury and that impairment of the Nrf2-p62 pathway is responsible for defects in autophagy in these melanocytes (39). Autophagy occurs in melanocytes from nonlesional skin of vitiligo patients as a result of the metabolic surveillance response (40). Thus, we believe that autophagy defects may play a central role in the regulation of HMGB1 release under oxidative stress conditions. Understanding the relationship between HMGB1 and autophagy in the context of cellular adaptation to injury and unscheduled melanocyte death is crucial.

As an endogenous danger signal, HMGB1 acts on its receptors, including several TLRs and RAGE, to initiate or amplify the subsequent inflammatory and immune response (41). In the perilesional skin of vitiligo patients, the expression of TLR2, TLR4 and RAGE on the surface of dendritic cells (DCs) is increased (36). Extracellular disulfide-HMGB1 binds to these receptors and induces DC maturation and activation, which activates melanocyte antigen-specific CD8^+^ T cells and thus initiates an autoimmune response in patients with vitiligo (36).

**Figure 2:**
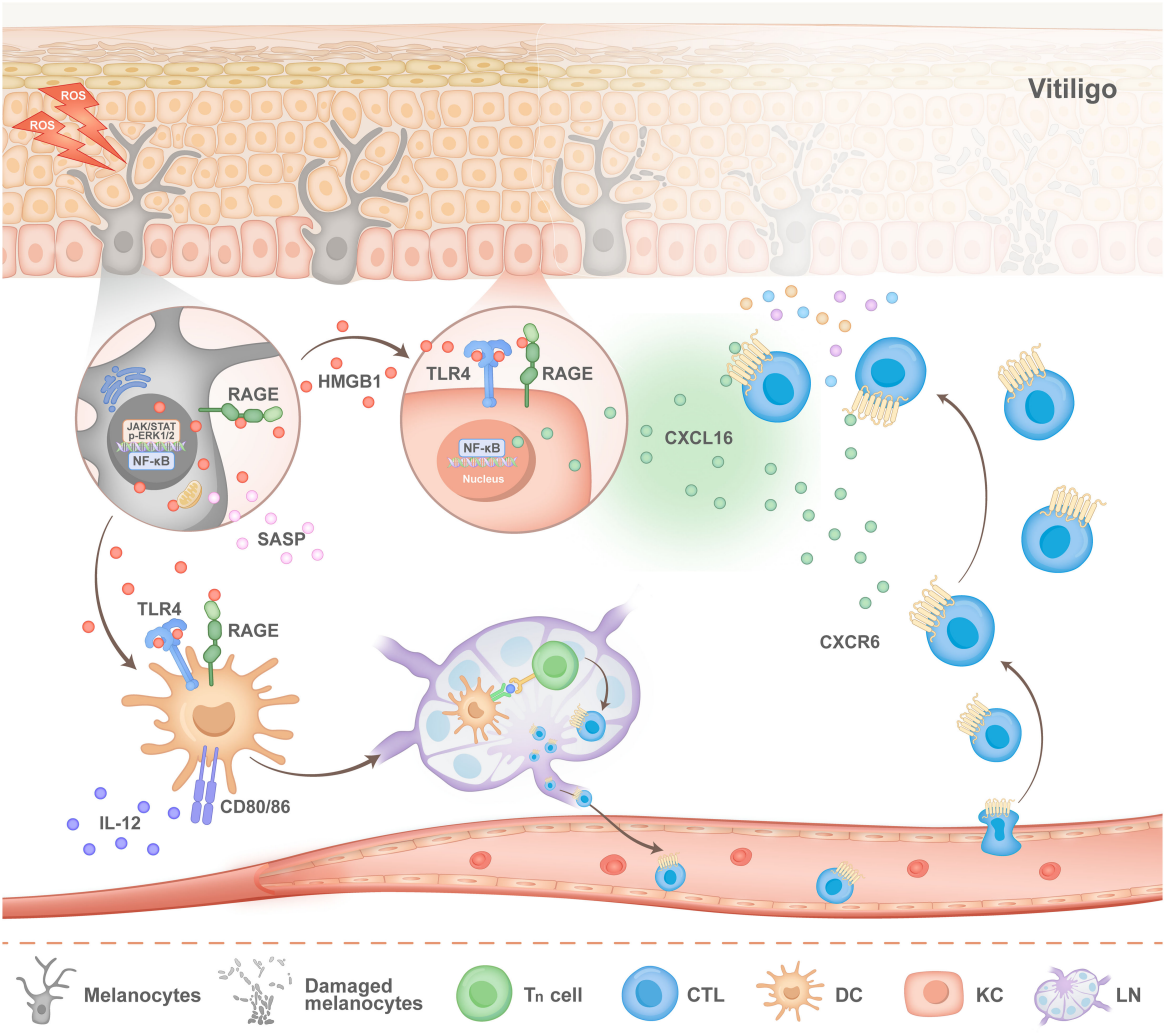
Pathogenesis of HMGB1 in vitiligo. Stimulated melanocytes (MCs) exhibit activation of the nuclear factor-κB (NF-κB) and JAK signalling pathways, both of which facilitate the nuclear translocation and subsequent release of HMGB1. Extracellular HMGB1 can bind to the receptor for advanced glycation end products (RAGE) on melanocytes, thereby activating the Janus kinase (JAK)/signal transducer and activator of transcription (STAT) signalling pathway and the extracellular signal-regulated kinase (ERK) pathway. This activation mediates the release of the senescence-associated secretory phenotype (SASP). On the one hand, HMGB1 binds to Toll-like receptor 4 (TLR4) and RAGE receptors on the surface of keratinocytes (KCs), which stimulates KCs to secrete CXC chemokine ligand 16 (CXCL16). On the other hand, HMGB1 binds to TLR4 and RAGE located on the surface of dendritic cells (DCs), which promotes the migration of DCs to lymph nodes and the activation of T cells. After activated T cells migrate from the lymph nodes into blood vessels, CXCL16 secreted by KCs recruits T cells to lesions and accelerates the progression of vitiligo disease.

In addition to melanocytes, oxidative stress can also induce keratinocytes to release HMGB1 (36, 42, 43). By binding to RAGE and TLRs on the surface of keratinocytes, HMGB1 affects melanocyte survival and the expression of molecules associated with melanin production, which is a newly discovered way in which keratinocytes influence melanocyte function. More importantly, HMGB1 activates NF-κB signalling and then promotes the production of chemokines, including CXC chemokine ligand 16 (CXCL16) and interleukin-8 (IL-8), thereby inducing CD8^+^ T cell migration and establishing an immune microenvironment unique to vitiligo (36) (**Figure 2**).

## HMGB1 in psoriasis

Psoriasis is a common, chronic papulosquamous skin disease that is characterized by excessive hyperproliferation and aberrant differentiation of keratinocytes and is caused mainly by the Th17/IL-23 immune axis. Compared with those in healthy donors, HMGB1 levels have been reported to be increased in the serum and skin lesions of patients with psoriasis vulgaris (PV), and while serum HMGB1 levels significantly increase with disease progression, they are downregulated after standard therapies are given (44, 45). Generalized pustular psoriasis (GPP) is a rare but severe variant of psoriasis, and the levels of HMGB1 in both the skin and serum are significantly greater in patients with GPP than in those with PV and healthy controls (46). Similarly, serum HMGB1 levels are significantly decreased after systemic treatment in GPP patients (46). These observations suggest that HMGB1 is significantly associated with psoriasis morbidity.

It has been reported that, in PV patients, increased HMGB1 is secreted from epidermal keratinocytes and some dermal cells (47) and that IMQ induces psoriasis-like inflammation (48–50). The abundant cytosolic and extracellular HMGB1 in lesional skin is usually associated with increased expression of its receptor, RAGE, on the cell surface of both keratinocytes and immune cells in patients with PV (47). In keratinocytes, extracellular disulfide-HMGB1 facilitates the expression of IL-18 through autocrine activation of the NF-κB p65 signalling pathway and the inflammasome; subsequently, in a mouse model of imiquimod (IMQ)-induced psoriasis, the HMGB1 and IL-18 secreted from keratinocytes contribute to IL-17 production in CD4^+^ T cells and the development of psoriasis by binding to RAGE (51). In lipopolysaccharide (LPS)-induced keratinocytes and IMQ-induced psoriasiform dermatitis in mice, once the translocation of HMGB1 from the nucleus to the cytoplasm is inhibited, the expression of RAGE, TLR4, p-ERK1/2, and nuclear NF-κB p65 as well as that of proinflammatory cytokines including IL-6, IL-1β, TNF-α, IL-17, IL-22 and IL-23, is significantly decreased (49). These studies suggest that HMGB1 can control the activation of various molecular signalling pathways thereby exerting a proinflammatory effect on psoriasis.

Notably, CD8^+^ T cells and CD4^+^ Treg cells in patients with PV also express increased levels of RAGE compared with healthy controls (47). In proinflammatory diseases such as rheumatoid arthritis, HMGB1 modulates the Treg/Th17 balance towards Th17 cells and thus enhances the Th17-associated pathogenic immune response (52); this may also occur in psoriasis since activated HMGB1-RAGE signalling and a Th17-dominated immune response function in the progression of psoriasis. A recent study demonstrated that HMGB1 can promote Th17 cell differentiation from PBMCs derived from psoriasis patients and can increase the production of its effective cytokine IL‐17A in a dose-dependent manner (53). These processes occur, at least in part, due to the binding of HMGB1 to the TLR4 receptor, which promotes the expression of retinoid-associated orphan receptor γt (RORγt, a Th17 cell-specific transcription factor) and IL-23 (Th17 differentiation associated key cytokine) (53). Th17 cell differentiation is also orchestrated by a network of cytokines, with IL-1β and IL-6 serving as pivotal molecular drivers of this process (54). Previous studies have indicated that HMGB1 can promote the secretion of IL-6 and IL-1β by monocytes, thereby sustaining the inflammatory response (55, 56). This may constitute an additional pathway by which HMGB1 regulates Th17 cell differentiation, which warrants further investigation.

**Figure 3:**
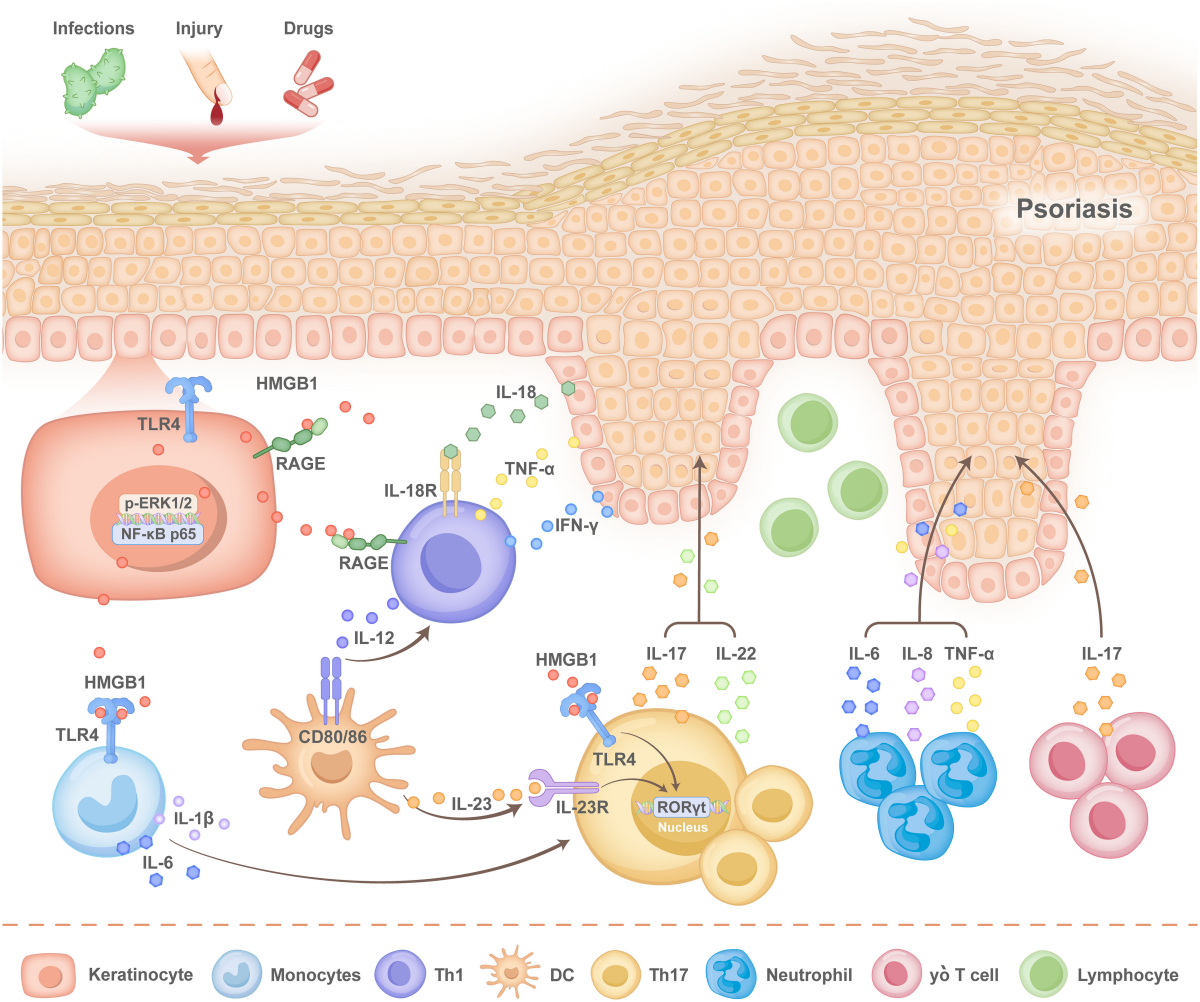
Pathogenesis of HMGB1 in psoriasis. When the normal epidermis of patients with psoriasis is subjected to external stimuli, such as infection, injury and drug stimulation, human high mobility group protein 1 (HMGB1), produced by keratinocytes (KCs), is released into the extracellular space via autosecretion, after which it activates the nuclear factor-κB (NF-κB) p65 signalling pathway. The KC inflammasome is activated to promote the secretion of interleukin-18 (IL-18) by KCs. Simultaneously, the translocation of HMGB1 from the nucleus to the cytoplasm activates the receptor for advanced glycation end products (RAGE), Toll-like receptor 4 (TLR4), and the phospho- extracellular signal-regulated kinase1/2 (p-ERK1/2) pathways, as well as the expression of pro-inflammatory cytokines such as interleukin-1β (IL-1β), interleukin-6 (IL-6), and tumour necrosis factor-alpha (TNF-α).HMGB1 and IL-18 bind to RAGE and interleukin-18 receptor (IL-18R) located on the surface of T cells, respectively, which promotes the secretion of interferon-γ (IFNγ) and TNF-α by T helper 1 (Th1) cells and that of IL-17 by T helper 17 (Th17) cells. The binding of HMGB1 to TLR4 on the surface of Th17 cells promotes the transcription of retinoid-associated orphan receptor γt (RORγt) and the expression of interleukin-23 (IL-23). Additionally, HMGB1 interacts with TLR4 on monocytes, which stimulates the secretion of IL-1β and IL-6. IL-1β and IL-6 further enhance Th17 cell differentiation, thereby sustaining the inflammatory response. Various cytokines lead to the aggregation of neutrophils, gamma delta (γδ) T cells and dendritic cells (DCs), which forms a positive feedback loop that accelerates epidermal thickness and disease progression.

In an IMQ-induced mouse model, skin injection of HMGB1 enhances the expression of multiple cytokines, including IL-6, IL-17, TNF-α, and interferon-γ (IFN-γ), and results in increased infiltration of CD3^+^ T cells, neutrophils, CD11c^+^ dendritic cells, and gamma delta (γδ) T cells (57). Previous studies have confirmed the crosstalk between KC-specific HMGB1-associated secretion and γδT cells in psoriasis (58) (**Figure 3**). These findings suggest that HMGB1 acts as an important proinflammatory cytokine and contributes to the balance of immune cells and the development of psoriasis.

## HMGB1 in atopic dermatitis

Atopic dermatitis (AD) is the most common chronic inflammatory skin disease and is often associated with different atopic and allergic comorbidities induced by overactivated type 2 immunity. AD pathogenesis is related to the interplay between defects in the skin barrier and immune dysregulation. Immune dysregulation characterized by increased numbers of Th2 cells, Th22 cells and Th17 cells plays an important role in the development of AD. In human skin, HMGB1 stimulation inhibits the expression of key structural proteins, including filaggrin and loricrin, thereby damaging the epidermal barrier (59), which may result in AD initiation. Compared with that in healthy controls, the serum HMGB1 level is significantly increased in patients with AD and is positively correlated with the severity of skin lesions, as indicated by the scoring atopic dermatitis (SCORAD) index (60); the serum levels of total immunoglobulin E (IgE), IL-17, and IL-23 are also increased in AD and are inversely correlated with the serum IL-10 level. These observations suggest that HMGB1 is an ideal biomarker for the severity of AD. Increased expression levels of cytoplasmic HMGB1 have been observed in both epidermal keratinocytes and dermal infiltrating immune cells in AD patients compared with healthy controls and those with psoriasis (61).

**Figure 4:**
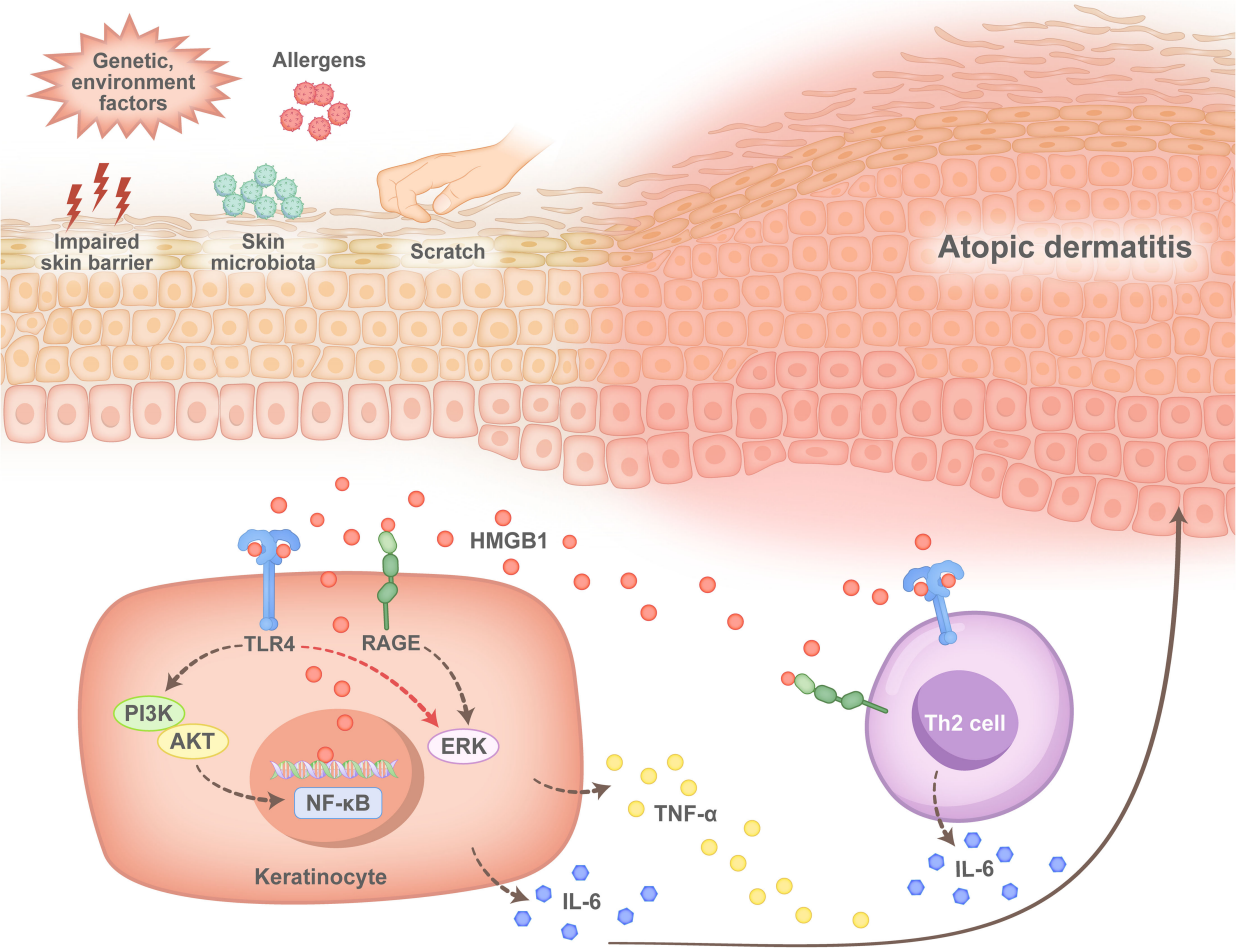
Pathogenesis of HMGB1 in atopic dermatitis. After the skin of patients with atopic dermatitis is damaged by impairment of the skin barrier, microbial infection or scratch, keratinocytes (KCs) produce HMGB1, which binds to Toll-like receptor 4 (TLR4) and the receptor for advanced glycation end products (RAGE) on the surface of KCs, after which the phosphoinositide 3-kinase (PI3Ks)/AKT serine/nuclear factor-κB (PI3K/AKT/NF-κb) pathway or the extracellular regulated protein kinase (ERK)/NF-κB pathway is activated. This leads to the production of the proinflammatory cytokines interleukin-6 (IL-6) and tumour necrosis factor-alpha (TNF-α). In contrast, HMGB1 binds to TLR4 or RAGE located on the surface of T helper 2 (Th2) cells, which promotes the activation and secretion of IL-6 by Th2 cells and promotes an immune response in patients with atopic dermatitis during disease progression.

Consistently, increased NF-kB activation signals in skin keratinocytes and immune cells in AD patients are significantly stronger than those in PV patients and healthy controls (61), which indicates increased HMGB1/NF-κB pathway activity in the skin of patients with AD. Studies have indicated that, in dermatitis, cytoplasmic HMGB1 activates the PI3K/AKT/NF-κB and ERK/NF-κB signalling pathways, which contributes to increased expression of HMGB1 and its receptors, such as RAGE and TLR4, as well as the subsequent production of proinflammatory cytokines, such as TNF-α and IL-6 (62). Since both RAGE and TLR4 are prominently expressed in Th2 cells in AD patients, we believe that extracellular HMGB1 interacts with RAGE or TLR4 and plays a critical role in the abnormal immune response in AD development (**Figure 4**). The pathogenic role of HMGB1 in AD has been confirmed in mouse models (62–65), but a more detailed mechanism requires further investigation.

## HMGB1 in alopecia areata

**Figure 5:**
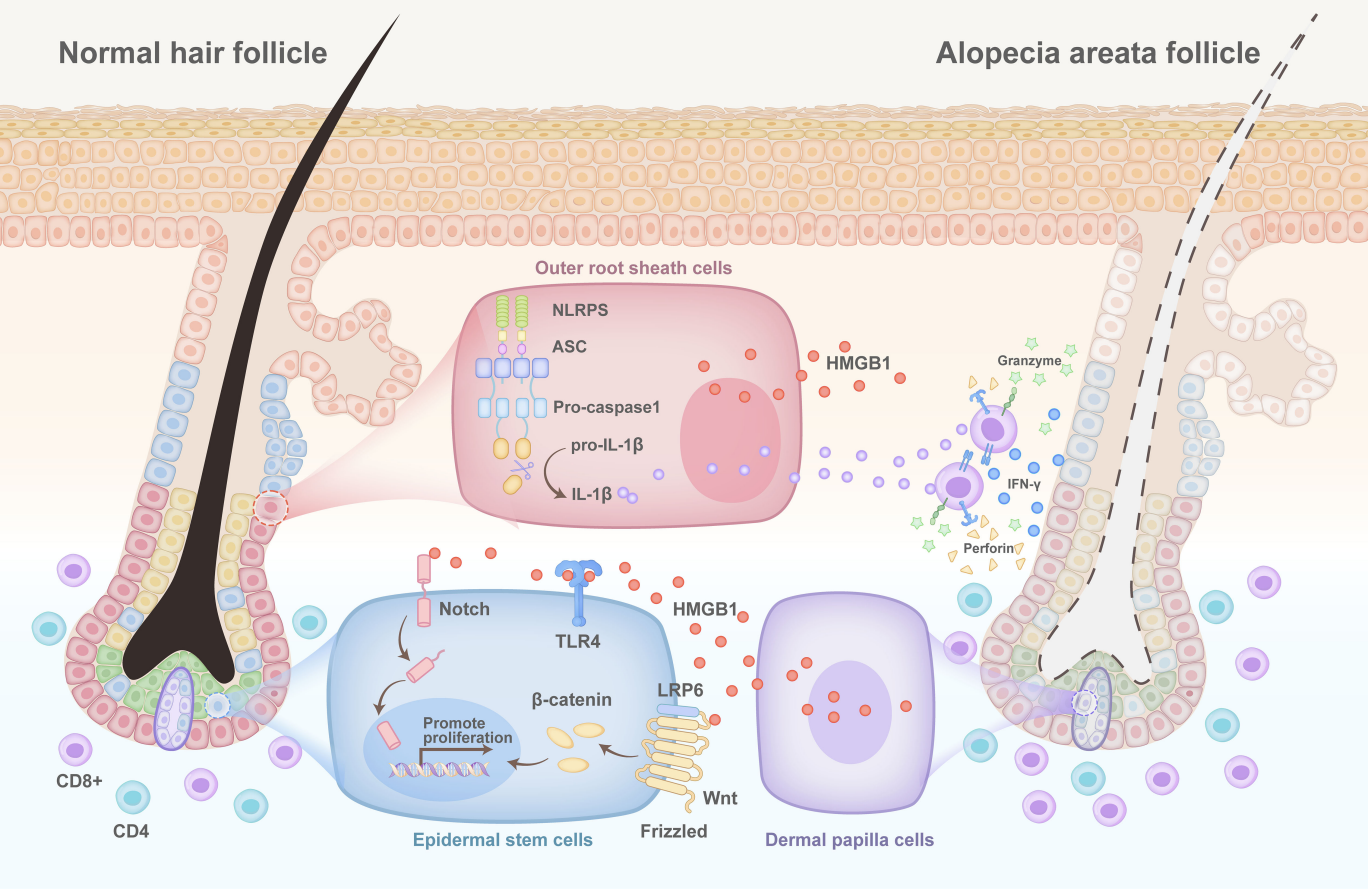
Pathogenesis of HMGB1 in alopecia areata. The NLR family pyrin domain-containing 3 (NLRP3) inflammasome is activated in the outer hair root sheath cells of patients with alopecia areata. This results in the simultaneous release of interleukin-1β (IL-1β) and high mobility group protein 1 (HMGB1), which bind to the interleukin-1 receptor (IL-1R) and the receptor for advanced glycation end products (RAGE)/toll-like receptors (TLRs) on the surface of CD8^+^ T cells, respectively, and promote the secretion of interferon-γ (IFNγ) by CD8^+^ T cells. Many CD8^+^ T cells accumulate to accelerate the death of hair follicles, while injured hair follicle dermal papilla cells secrete HMGB1. This activates the WNT/Notch signalling pathway through binding to TLR4 on the surface of stem cells to promote their proliferation, thereby regenerating hair.

Alopecia areata (AA) is a T cell-mediated autoimmune disease characterized by chronic and relapsing hair loss. Serum HMGB1 levels are elevated in patients with AA and are associated with disease severity. The serum HMGB1 level in patients with alopecia universalis is much higher than that in patients with patchy alopecia, alopecia totals and healthy controls and is considered an independent predictor of AA severity (66, 67). Moreover, it has been reported that increased serum HMGB1 levels are correlated with poor treatment responses (66). Histological examination revealed substantially increased expression of HMGB1 in the dermis of scalp tissues from patients with AA (66). Further studies revealed that, in the outer root sheath cells of hair follicles, HMGB1 is transported from the nucleus to the cytoplasm and then released into the extracellular space in response to stimulation with the double-stranded RNA mimic polyinosinic:polycytidylic acid. The secretion of HMGB1 is controlled by NLRP3 inflammasome activation in outer root sheath cells, which suggests that HMGB1 collaborates with IL-1β, an effector of the NLR family pyrin domain-containing 3 (NLRP3) inflammasome, to promote inflammation in patients with AA (68). However, in ex vivo hair organ culture, HMGB1 significantly increased the expression and secretion levels of prostaglandin E from dermal papilla cells via interaction with its canonical receptor, RAGE and thus increased hair shaft elongation (69). Importantly, in damaged tissues, extracellular HMGB1 can accelerate wound healing by promoting the differentiation of epidermal stem cells through the “HMGB1-TLR4-Wnt/Notch” axis (70) (**Figure 5**). These results suggest that HMGB1 can also promote hair growth and tissue regeneration in response to damage. The detailed role of HMGB1 in the pathogenesis of AA is far more complex than is currently known. We speculate that the dual role of HMGB1 in AA may be stage-specific and microenvironment-dependent and is related to its cellular origin as well as intercellular communication. Specifically, during the rapid progression of AA, HMGB1 secreted by damaged follicle outer root sheath cells may play a proinflammatory role mainly by enhancing the activation and function of T cells. However, in the stable phase of AA, when the immunoinflammatory response is weakened, HMGB1 secreted by dermal papilla cells may interact with receptors that on stem cells in hair follicles, thereby promoting hair regeneration. We hypothesize that extracellular HMGB1 is likely an important molecule involved in the balance and regulation of the “proinflammatory microenvironment-tissue repair and regeneration” cycle in AA.

## HMGB1 in other autoimmune skin diseases

Polymyositis (PM) and dermatomyositis (DM) are the two major inflammatory muscle diseases characterized clinically by proximal muscle weakness. High cytoplasmic and extracellular expression levels of HMGB1 have been consistently detected in muscle fibres, endothelial cells and mononuclear inflammatory cells of PM/DM patients in the early phase of their disease (71, 72). Serum HMGB1 levels are also elevated in patients with PM/DM (73). Studies have indicated that IFN-γ increases both the nuclear and the myoplasmic expression of HMGB1 in adult skeletal muscle fibres. Moreover, studies have confirmed that HMGB1, an important mediator, can drive MHC class I (MHC-I) expression in muscle fibres by binding to TLR4 but not to RAGE (72, 74). Given that an increase in MHC-I in muscle fibres is sufficient to trigger muscle inflammation and weakness in animals (75), HMGB1 is considered a necessary initial step in inducing PM/DM onset and directly contributes to muscle weakness.

The impaired regeneration capacity of muscles is also responsible for the development of muscle weakness in patients with PM/DM. Notably, lower HMGB1 expression is accompanied by impaired proliferation in primary muscle cell cultures obtained from patients with PM/DM compared with cultures of healthy muscles affected by coxarthrosis muscles (76). In addition, another study showed that extracellular HMGB1 could improve the differentiation of and attenuate the proliferation of rat myoblasts through its interaction with RAGE (77). These findings suggest that HMGB1 may also be involved in the progression of PM/DM by affecting the proliferation and differentiation of muscles, which requires further investigation. Another potential mechanism by which HMGB1 contributes to PM/DM progression is the induction of muscle dysfunction (74). Although HMGB1 can increase autophagy in muscle fibres (78) and thus facilitate the clearance of damaged cellular components under certain conditions, in the case of PM/DM, it can also lead to excessive degradation and functional loss of muscle fibres (79). Furthermore, HMGB1 can activate inflammatory signalling pathways, such as the NF-κB pathway, to promote the release of IL-1β, TNF-α, and IL-6, thereby exacerbating inflammatory responses and tissue damage in muscle (71, 80, 81). PM/DM can also present with systemic manifestations, specifically a significant increase in interstitial lung disease (ILD), which is an important complication that affects disease prognosis. Serum HMGB1 is overexpressed in new-onset PM/DM patients with ILD; patients with higher serum HMGB1 levels have lower overall survival, whereas those with lower serum HMGB1 levels have higher overall survival (82). This finding suggests that HMGB1 is a prognostic indicator of disease progression and helps predict poor outcomes.

Pemphigus is a life-threatening autoimmune skin disease characterized by blistering skin and/or mucous membranes induced by antibodies that bind to desmoglein (DSG) 3 and 1. It has been reported that serum HMGB1 levels are clearly increased in pemphigus patients and are significantly decreased after treatment. In skin samples, increased cytoplasmic expression of HMGB1 and its receptor RAGE was observed in the epidermal keratinocytes of patients with pemphigus (83, 84). In patients with bullous pemphigoid, neither the serum HMGB1 levels nor the nuclear HMGB1 expression in the skin are significantly different from those in healthy controls (83). Therefore, we suggest that the HMGB1/RAGE interaction may contribute to inflammatory reactions and tissue damage in the pathogenesis of pemphigus but not that of bullous pemphigoid.

## HMGB1-targeting therapies

Several HMGB1-directed therapies are under investigation (**Table 1**), although none are yet approved for dermatological indications. Glycyrrhizin (GL), a natural triterpene derived from liquorice root, directly binds to the A- and B-box domains of HMGB1, which inhibits its interaction with RAGE and TLR4 (85). GL, which is approved in Japan for the treatment of chronic hepatitis, reduces serum HMGB1 levels and alleviates inflammation in viral dermatitis models (86), but its long-term use is limited by dose-dependent hypertension and hypokalaemia (87). Monoclonal antibodies (mAbs) offer increased specificity: 2G7 (targeting the A-box) and DPH1.1 (targeting the B-box) show efficacy in experimental arthritis models and viral hepatitis (88, 89), although human trials are pending. Notably, SB17170, an oral prodrug that is metabolized to the HMGB1 inhibitor SB1703, is in phase I/II trials as a treatment for solid tumours (NCT05795192; NCT05522868) and therefore has potential dermatological applications. The recombinant A-box (BoxA) protein acts as a decoy receptor to suppress HMGB1-mediated inflammation in arthritis models, but its mechanism is not completely defined, and immunogenicity concerns persist (90). Soluble RAGE (sRAGE), a natural HMGB1 antagonist, reduces arterial calcification by inhibiting the AT1R-HMGB1-RAGE axis (91). Emerging strategies include the peptide P5779, which disrupts HMGB1-TLR4/TLR4 binding without affecting pathogen-associated TLR4 activation (92). The adverse effects common across these therapies include transient immunosuppression and interference with the physiological roles of HMGB1 in DNA repair and autophagy (93).

**Table 1:** HMGB1-targeting therapies in clinical development progress.

Therapy	Mechanism of Action	Current Status	Efficacy	Limitations/Adverse Effects	References
Glycyrrhizin (GL)	Binds A- and B-box domains of HMGB1, inhibiting interaction with RAGE and TLR4	Approved in Japan for chronic hepatitis; tested in viral dermatitis models	Reduces serum HMGB1 levels and alleviates inflammation	Limited long-term use for dose-dependent hypertension, hypokalaemia	(85) (86) (87)
Monoclonal Antibodies	2G7 (targets A-box); DPH1.1 (targets B-box)	Experimental arthritis models and viral hepatitis; human trials pending	Effectively improved arthritis in mouse models	Pending human trial data	(88) (89)
SB17170 (Prodrug)	Metabolized to HMGB1 inhibitor SB1703	Phase I/II trials for solid tumours (NCT05795192; NCT05522868); potential dermatological applications	Promising in early clinical trials	Adverse effects not fully characterized; potential immunosuppression	(NCT05795192; NCT05522868)
Recombinant A-box (Box A)	Acts as a decoy receptor, suppressing HMGB1-mediated inflammation	Tested in arthritis models	Effective in preclinical inflammation models	Mechanism incompletely defined; immunogenicity concerns	(90)
Soluble RAGE (sRAGE)	Natural HMGB1 antagonist; inhibits AT1R-HMGB1-RAGE axis	Preclinical studies; reduces arterial calcification	Reduces inflammation and calcification in preclinical models	Limited clinical data	(91)
Peptide P5779	Disrupts HMGB1-TLR4 binding without affecting pathogen-associated TLR4 activation	Preclinical studies	Reduces mortality in rabbit and rodent models of hepatic ischaemia/reperfusion injury, chemical toxicity, and sepsis	Early-stage research	(92)

## Bridging the gap between preclinical findings and clinical applications

Despite promising preclinical evidence implicating HMGB1 as a therapeutic target in autoimmune and autoinflammatory skin diseases, significant challenges have impeded its clinical translation. A key hurdle lies in the pleiotropic role of HMGB1, which varies depending on its redox state, subcellular localization, PTMs, and interaction partners. For example, extracellularly reduced HMGB1 exhibits chemotactic activity, while disulfide HMGB1 activates proinflammatory cytokine release via TLR4 signalling, and fully oxidized HMGB1 is inert or even anti-inflammatory (94, 95). Emerging evidence suggests that PTM-specific isoforms, including acetylation, phosphorylation, and methylation variants, affect the secretion and release of HMGB1 (1, 96). This redox- and PTM-dependent functional dichotomy complicates therapeutic targeting, as broad HMGB1 inhibition may inadvertently be disrupted. Its homeostatic roles include tissue repair and immune regulation (95).

Furthermore, HMGB1 inhibitors often lack specificity: indirect inhibitors such as ethyl pyruvate exhibit pleiotropic anti-inflammatory effects by suppressing the NF-κB and inflammasome pathways, which raises concerns about off-target consequences (97). Even direct inhibitors, such as glycyrrhizin (GL), bind HMGB1 with moderate affinity (Kd ∼150 μM), and thus they may also affect structurally similar proteins. Future efforts to design isoform-selective inhibitors should focus on identifying PTM “hotspots” that differentiate pathogenic HMGB1 variants from their physiological counterparts.

Clinical translation is further hampered by the flexible, solvent-exposed structure of HMGB1, which lacks deep hydrophobic pockets for high-affinity drug binding (85). While preclinical models of lupus and atopic dermatitis have shown the efficacy of HMGB1 blockade (62, 64, 98), dermatology-specific clinical trials are still lacking. No HMGB1-targeted therapies for skin diseases have advanced to clinical trials, which is partly due to uncertainties about dosing, long-term safety, and biomarker-guided patient stratification. Standardized assays to quantify extracellular HMGB1 isoforms, including PTM-specific variants, are urgently needed to stratify patients based on their dominant HMGB1 pathogenic profile. Addressing these gaps requires optimized inhibitors with redox-state specificity, standardized assays to quantify extracellular HMGB1 isoforms, and rigorously designed trials that evaluate both the efficacy and risks of immunosuppression.

## The challenges of HMGB1 as a biomarker

HMGB1 has emerged as a promising biomarker in autoimmune and autoinflammatory skin diseases, as accumulating evidence links its disease-specific expression patterns to inflammatory cascades in conditions such as vitiligo, psoriasis, atopic dermatitis, and pemphigus. Released by epidermal keratinocytes and immune cells, HMGB1 activates innate immunity through the TLR4/NF-κB pathway, which is correlated with disease flares and severity. Clinically, reductions in serum HMGB1 levels parallel therapeutic responses to systemic treatments, which supports its utility in monitoring skin disease prognosis. However, challenges persist, including its limited specificity as a standalone biomarker due to the shared increase in infections and malignancies, which necessitates combinatorial strategies with disease-specific markers like IL-17 for psoriasis (99, 100). Technical limitations have also hindered progress, as current ELISA-based assays exhibit interlaboratory variability, particularly in distinguishing different HMGB1 isoforms (nonacetylated vs. acetylated HMGB1) (93). Large-scale clinical validation remains constrained by the lack of standardized protocols and reproducible thresholds, which emphasizes the need for harmonized detection methods and multiomics integration to advance HMGB1 from its current position, where mechanistic insights are known, to its use as an actionable clinical tool.

## Conclusions and perspectives

HMGB1 has distinct functions inside and outside the cell. Over the past 20 years, increasing numbers of studies have focused on the role of extracellular and cytoplasmic HMGB1 in cellular stress and immune responses, and it has been confirmed that extracellular HMGB1 is a risk factor for many inflammatory and autoimmune skin diseases. Elevated serum HMGB1 levels are positively correlated with the activity or severity of multiple skin diseases. Therefore, HMGB1 can be a useful biomarker for the diagnosis and prognosis of various inflammatory skin diseases. HMGB1 antagonists have shown great success in multiple preclinical animal models of autoimmune diseases, which suggests that HMGB1 is an attractive therapeutic target. Thus, further exploration of strategies that combine HMGB1 with other targets is highly important for the treatment of skin diseases. Although HMGB1-related research still faces many challenges, we anticipate future studies that will maximize the potential of HMGB1 as a therapeutic target.
